# Supplementary material for: Cataract Surgery in Low-Income Countries: A Good Deal!
Source: Healthcare (Basel). 2022 Dec 19;10(12):2580. doi: 10.3390/healthcare10122580 (PMC9778186; doi:10.3390/healthcare10122580)
Supplement: Supplementary file 1 [file healthcare-10-02580-s001.zip › healthcare-2020967-supplementary.pdf]

Prof. Dr. Steffen Fleßa

UNIVERSITÄT GREIFSWALD
Wissen lockt. Seit 1456

Questionnaire Cataract Surgery in Africa

Dear colleagues, first calculations show that Cataract surgery in low-income countries is cost-saving or highly cost-effective. However, the main limitation of these computations is the low reliability of economic data. Most data exists for high-income countries, but it is questionable whether it is applicable for low-income countries. Other data is obsolete.

Therefore, I would like to ask for your support giving your estimates of epidemiological and economic data. I would like to focus on a rural district in Sub-Saharan Africa. Please provide me with your best estimates for averages based on your experiences. If you are not sure, please give your best estimate. We will analyse all questionnaires and give back to you the results. If you want, you can adjust your estimates afterwards (2-Stage-Delphi).

1. In which age is on average the on-set of Cataract (mild impairment according to WHO)?
   - In the age of ____ years.
2. What is the duration of the following stages?
   - Mild and moderate impairment ____ years
   - Severe impairment ____ years
   - Blindness (until death) ____ years
3. When is the best time to perform Cataract surgery in the above-mentioned context?
   a. With onset (mild impairment) ◯
   b. With beginning of severe impairment ◯
   c. With beginning of blindness ◯
   d. Else: ____
4. How do you assess the following costs in a rural district in Sub-Saharan Africa?
   a. Treatment cost of Cataract patients per year (consultations, medication, glasses, …; but without surgery cost)

      _______________ US$ per year per person

   b. Surgery cost of Cataract (both eyes, incl. post-surgical care)
      _______________ US$ per person
5. How do you assess the reduction of the work productivity of a patient with Cataract?
   a. Mild impairment: ____ %
   b. Severe impairment: ____ %
   c. Blindness: ____ %
6. How much time must a care-giver invest to take care of a person suffering from Cataract?
   a. Mild impairment: ____ hours per day
   b. Severe impairment: ____ hours per day
   c. Blindness: ____ hours per day

Thank you very much! If you would like to receive the results of the survey and participate in a second stage, please give me your E-Mail address, otherwise just leave it blank, but please hand-over the filled questionnaire to me.

E-Mail: ________________________@____
